# Obtaining a faculty position in STEM at a research-intensive institution

**DOI:** 10.1186/s12919-021-00210-x

**Published:** 2021-06-22

**Authors:** Jim O. Vigoreaux, Michael J. Leibowitz

**Affiliations:** 1 Department of Biology and Office of the Provost, University of Vermont, Burlington, VT USA; 2 Department of Medical Microbiology & Immunology, University of California, Davis, CA USA

**Keywords:** Transition, Faculty, Research intensive institution

## Abstract

Progressing from postdoctoral training to a STEM faculty appointment at a Research Intensive Institution (RII) is a daunting transition, and may be especially challenging to those who have followed a less-than-conventional path or whose peers have lost interest in academic careers. This article describes how to prepare for and progress through the application process for institutions in the USA, which takes approximately 1 year, including what to expect at each step and recommendations for a successful transition. The odds of success for any individual application are low, making good preparation and careful planning the more important, as does managing expectations to avoid becoming discouraged early in the process. The rewards of landing the faculty appointment at an institution that matches your professional and personal needs and for which you are best suited more than exceeds the effort required to attain it.

## Background

The postdoctoral years are generally characterized as a mixture of joy and fear, with stretches of inspired productivity that soars confidence to new heights, to dry spells that shake one’s confidence, especially as the clock ticks towards the end of the post-doctoral funding period. Contemplating the enormity of the challenge may elicit periods of self-doubt, and these are likely to more common among individuals that have “made it” thus far via less-than-conventional path or whose many peers lost interest in academic research careers, namely underrepresented minorities URM [1, 2]. Through your graduate and post-doctoral years you have listened, with horror, to stories of the competitiveness of the market for a faculty position at research intensive institutions (RIIs) and the oversupply of PhDs vying for faculty positions [3]. By now you are familiar with the accounts of hundreds of applications for one position, of individuals with enough rejection letters to wallpaper their offices and relegated to seemingly endless cycles of precarious post-doctoral and adjunct positions [4]. You read with dismay how the Covid-19 pandemic has exacerbated an already challenging situation as college after college, driven largely by reduced student demand, slash their budgets, reduce or freeze their faculty hires, issue broad furloughs, terminate tenured faculty or close altogether [5–7]. Yet, you also read about the increasing trend in faculty positions [8], the dire need for innovation to address earth’s and humanity’s pressing challenges [9–11], the depleted scientific workforce and the increasing demands for STEM professionals [3], how philanthropies and private donors are stepping up their support for science [12, 13], how federal funding levels for research remains on a robust ascending slope [14–16], and how universities are building up their STEM enterprises [17]. You remain hopeful and upbeat knowing that with challenge comes opportunity and that in the roller coaster that is the economy every downturn is followed – eventually - by an upturn. The opportunity to fulfill your aspiration of a faculty position that contributes to knowledge creation and scientific advancement is awaiting you. You embrace the challenge of the competitive market knowing that landing your dream faculty position opens the door to a fulfilling and rewarding career.

Here, we provide a short guide to assist you in this important quest. We describe how to go about choosing, applying, interviewing, negotiating, transitioning, and getting started in your faculty position at a RII in the United States. The advice presented here emerges from a combination of the authors’ experiences at various types of RIIs, the work of the American Society for Cell Biology (ASCB) Innovative Programs to Enhance Research Training [18] and material from a 3 day *Accomplishing Career Transitions* workshop. Other articles in this series complement and expand on some of the topics covered here. We hope this article (and others in this series) inform your career transition plan and that it serves to highlight the importance of identifying reliable mentors and career confidants, establishing a robust professional network, and maximizing the career enhancing opportunities offered through your professional organizations and other public, private and government entities.

For those seeking appointments in non-U.S. institutions, the general approach will be similar to that described here, but the specifics vary greatly among countries and institutions in a single country. The recruitment cycle in most foreign institutions starts with advertisements placed a bit later in the year than in the US.

### Know the options

One of the first decisions you face as a post-doc is the type of academic institution to which to apply. These fall largely into two categories based on their emphasis on teaching or research: primarily undergraduate institutions (PUIs) and RIIs. Advice for PUI positions is offered in the accompanying article by King-Smith et al. [19]. PUIs and RIIs are not polar opposites as research is expected at most PUIs and teaching is expected at most RIIs. Your decision rests on which of these two major areas you can be most impactful as a scientist, and which gives you the greatest satisfaction.

Intuition and common wisdom dictate that to land a position at a RII your post-doctoral years must be fully devoted to research, authoring publications, building collaborations, and obtaining grant funding. While these are certainly at the forefront, is that all that is needed? The answer is largely No as there are other aspects to the job. How to balance your preparation and gain the experience that will make you competitive depends largely on the type of university to which you apply.

A good start is to examine the Carnegie Classification of Institutions of Higher Education [20]. RIIs are found in Basic Classification. Based largely on the number of doctorates conferred and total research expenditures, Carnegie assigns a research activity index of R1 (very high research activity/highest research activity) and R2 (high research activity/higher research activity). There are approximately equal numbers (~ 130) of R1 and R2 institutions. A third category, Doctoral/Professional Universities, is assigned to institutions with less than $5 million in research expenditures or conferred fewer than 20 research and scholarship doctorates.

While each institution is unique, there are broad underlying similarities. Institutional reputation and geographical location figure prominently in the choice of where to apply but there are numerous other factors that are equally important. Your key tenet in a job search is what institution offers you the best opportunity for your professional development and achievement of career aspirations, and accomplishing the teaching and scholarship goals you have set for yourself. There are numerous factors that should weigh in your decision making. Generally:

- Broad spectrum of research-intensive institutions -
  - Small private to large land-grant public
  - Institutions that only offer graduate programs in specific disciplines, are STEM-focused, and those that offer graduate programs in many STEM and non-STEM disciplines
- Medical (health science/medical complex) and other professional schools on the same campus, for research that overlaps with disciplines in professional degree programs (e.g., Education, Health Professions).
- Private institutions tend to offer higher salaries; public institution faculty tend to be represented by unions or faculty organizations and this potentially provides more protection to your employment. Some public institutions with medical or other professional schools may have a combination of both represented and non-represented faculty ranks. Check the details of each school in terms of labor relations, since there is a lot of individual variation.

A note of R1 vs R2. You are likely to find a strong research culture and state-of-the-art infrastructure at R2 that due to their size do not meet the criteria for R1. These institutions present ideal homes for those who prioritize familiarity with their surroundings, a more intimate institutional culture and environment, and a sense of community belonging.

### Know the expectations

Faculty positions at a RII can go by different names based on the main job responsibility. These generally but not always are referred to as tenure track, research track, and education track. Knowing the options available at the institution is important as they differ in terms of workload expectations, pay, length of contract and job security, and access to students. A key difference between research track and tenure track, at most institutions, is that the former carries an expectation of providing part (or all) of the salary from grants and contracts and carries no long-term job security. But, research track positions afford the opportunity to dedicate 100% effort to research and salaries tend to be higher, as long as you are successful in obtaining funding.

For tenure track positions, the academic unit in which the department or program is housed also influences the nature of the position responsibilities. Most notably are the differences between departments in medical colleges and undergraduate colleges given the emphasis on undergraduate education of the latter. Teaching loads in medical college departments tend to be less in quantity and in more specialized subject matters than teaching loads in undergraduate colleges. It is not uncommon for a faculty member in an undergraduate department to teach across a range of levels – from introductory courses for non-majors to specialized courses for graduate students – and in different sub-specialties. The latter is especially true if the department is housed in a college of arts & sciences, where it might be the single department representing the biological sciences, in contrast to a department housed in a college of science, where there might be separate departments in related and over-lapping disciplines (e.g., biochemistry, cellular and developmental biology).

It is important to know the specific expectations for the position you are applying for. In most comprehensive universities, faculty are expected to teach regularly. One entire course per semester, or the equivalent, in addition to graduate seminar courses is a common practice. Such schools generally pay 100% of salary for 9 months (the academic year), with the expectation that you will seek grants or other sources to cover the 3 months of summer salary. You can choose to pay yourself less than the three full months and use funds to support students or postdocs. Also, at such schools much of the scientific workforce is made up of graduate students, which may be especially attractive if you enjoy mentoring at this level. Tenure at these institutions generally guarantees 9 months of salary, even if the faculty member is not grant supported.

In contrast, at most medical schools teaching may only consist of a few lectures per semester, plus graduate seminars. This leaves a larger fraction of faculty time free for research. On the other hand, there is a great variance in the salary provided among institutions and even among faculty at each one. Some faculty lines may pay all or most of the salary (especially at public institutions), while others may only do this for the initial period of employment, after which faculty and expected to pay all or most of their salary from grants. While tenure guarantees employment, it does not guarantee full salary and only that portion not expected to be covered by grants. In both medical schools and research institutes, most research is done by postdoctoral fellows rather than graduate students.

The institution’s sabbatical policy is a good thing to know about each appointment. Most comprehensive universities grant faculty one semester or a full year of sabbatical every 7 years. This usually requires an application that includes a research plan (e.g., a stay at someone else’s lab to learn a new technique) and must be approved by the department chair, dean or provost. The amount of sabbatical salary that is paid by the institution can range from zero to 100%; 50% is typical with the expectation that grants or other sources make up the rest. Some institutions allow one to shift all teaching for the year into one semester to extend a one semester sabbatical into two semesters.

Some positions offer the possibility of joint or secondary appointments, be it in a separate department or in a broader, interdisciplinary program. While these may entail an additional responsibility, they also open new opportunities (e.g., access to students from another program, departmental or shared facilities, and clinical research resources, among others).

Lastly, there are differences in workload expectations for the different tracks and even within the same track. For example, a tenure track position at one institution may have an expectation that a relatively high percentage of the effort (> 40%) be dedicated to research while at another institution, or even another department within the same institution, the expectation may be twice or half as much. These percentages may be negotiable within a certain range; we will discuss this in the next section.

One additional type of RII is the free-standing research institute, which is often very prestigious. Such institutions vary widely in how much of the salary is guaranteed with appointment and tenure, but at most it is expected that most or all of the salary comes from grants, and tenure may not have much meaning without such funding.

### Strategy and steps towards a career at research intensive institution

#### Address the gaps

You have been proudly and meticulously building up your *curriculum vitae* since your early college days. You have documented every activity, award, course and other information that portrays your training and talents and paints the arc of your *course of life*. As discussed earlier, faculty positions at RIIs come with many expectations in research, teaching (sometimes includes advising), a variety of service activities, and leadership. Now is the time to examine your *CV* through a different lens – what are the gaps in each of the three big categories of teaching, research, and service and how can you address them? We will examine these separately, but first a word of caution. Your approach to filling the gaps should not be one out of desperation or vanity, but rather carefully planned and strategic. Engage your mentors as much as possible.

Even at a RII there are expectations for excellent (and, in many cases, substantial) teaching. Graduate teaching assistant experience is now a baseline to build from during your post-doctoral years. Having taught as a graduate student will not separate you from the pack. Consider volunteering guest lectures in your program (e.g, filling in for a faculty member when they are traveling). Local schools and community colleges are places to look for paid or volunteer opportunities. Many job applicants list their experience supervising undergraduate or graduate students in their PI’s lab. Here you have an opportunity to communicate the effectiveness of that mentoring experience by describing what the student was able to accomplish during the supervised training period, or what the student went on to accomplish (e.g, presentation at a national conference, admission to a graduate program, co-author on your publication). Other ways to demonstrate your instructional chops include tutoring, creating short video tutorials of a technique you have perfected, or of a complex concept you can explain confidently. Talks at local and regional meetings and national conferences also contribute and help to get you known by potential mentors or colleagues at other institutions.

Various institutions offer boot-camp type programs for individuals contemplating academic careers. One variation of this is *Preparing Future Faculty* [21]. Find out if your current institution participates in this movement and how you can benefit from participating. Through this program you can gain experience at a partner institution different from yours, e.g., a community college or liberal arts college.

One of the unintended, and in the eyes of many positive consequences of the Covid-19 pandemic is that it accelerated the mastery of online and remote instruction and accelerated changes in higher education that were long overdue [22]. Many institutions invested substantial capital in training their instructors and upgrading their hardware and software in an effort to make online instruction feel like the real classroom experience. The need to balance the interest of students whether to attend classes remotely from home or in person on campus and the health realities of the location led to a variety of new teaching modalities that combine remote and in-person instruction. These add to the already varied menu of teaching and learning practices that are inclusive of all students and enhance the educational experience [23]. In a post Covid-19 world, experience in remote instruction is a quality many programs will expect of their faculty.

Tap into the resources of your institution’s Center for Teaching to gain familiarity with instructional technology and pedagogy. Attend workshops, discussions or online tutorials. Many of these are recorded and you can “attend” at your leisure. Follow instructional groups in LinkedIn or Facebook and become conversant in the latest instructional trends.

Research has been at the center of your career development since your early days in graduate school. Now that you are in the later stages of post-doctoral training and eyeing an independent research career, what steps must you take in blazing your own path? Two key questions are what would be the research project that will define your early independent career and what resources are needed to achieve it. The first question entails a conversation with your post-doctoral advisor, as well as the opinion of trusted colleagues, collaborators, and mentors. The same individuals can be useful sources of information for the second question that additionally engages your future supervisor (i.e., department chair, program director or dean) during your appointment negotiation.

With your post-doctoral advisor, decide projects and research questions that will be finalized by your departure date, those on which you will continue to collaborate and those that you will independently pursue. It is important to have these clearly identified as you begin the job search and reflected in the statement of research interests submitted as part of the application. There should be a clear path to an independent career while gradually (or completely) shedding off the role of your post-doc advisor. The research statement is the vehicle to lay down research problems you have identified and the approach you have designed to solve them. Launching your independent career seamlessly and quickly will play to your advantage in multiple ways. This is more easily achieved with projects that rely on your acquired skills, expertise, and collaborations. Projects that require a new skill set and training should be secondary on your list.

The third proverbial leg of the professoriate profession is service. Here you have an opportunity to showcase the causes that you deeply care and are passionate about and how they relate to your professional development and career aspirations. As a faculty member, the service that “counts” toward promotion and tenure is related to the institution and to the profession, e.g., membership in a committee of your professional society, organizing a science fair at your local elementary school. Service towards issues at the forefront of the national dialogue demonstrates your engagement as a committed citizen scientist. Topics that are particularly prescient as of this writing include, but are not limited to, the future of higher education in light of the pandemic, the downturn of the economy and changing demographics of college bound generation; race relations, equity and social justice; trust in science; community health (access to affordable health care) and environmental health (sustainability and climate change); globalization. Other types of service that are not directly related to education and your professional research field, while less important, can help demonstrate the values and principles that guide you as an individual and present an additional dimension your will bring to the institution. However, avoid having too much “busy work” service that does not strengthen your overall merit on paper.

#### The warm-up

While opportunities in academic science are plentiful the market is nevertheless highly competitive. Making the decision to accept employment at an academic institution is not only a critical career decision but potentially a lifetime decision. Faculty retention rates have been consistently high and the median duration of initial appointment of STEM academic faculty is between 10 and 11 years [24].

Among those that change institutions, faculty that start at a research institution are more likely to move to another research institution [25]. Thus, you want to survey the landscape of opportunities as thoroughly as possible and be well prepared to land the career of your dreams at a location or type of institution you would happily call home.

Many institutions seek to fill new faculty positions to coincide with their academic year. For schools that operate on a two-semester academic year, the usual start date is shortly before the beginning of the Fall semester in August or September. Position advertisements tend to peak in late summer/early fall, with interviews and selection late fall-early spring with most decisions before end of academic year but sometimes into the summer. Medical schools tend to recruit throughout the year. Look at a few advertisements and get a sense of their timelines. Regardless, plan for the process to consume a good part of 1 year. Beginning a “soft search” at least 2 years in advance of your anticipated move would serve this purpose.

A soft search consists of collecting information on the various types of institutions, faculty positions, and academic and research programs and how these intersect in defining the position. It also consists of information on the geographical location and local cultural, civic and economic forces that shape the locale. Good sources for this information are social media, print media, and your networks. Expand your social network to get a broad perspective on how individuals portray their institutions and how the institutional current situation and priorities are portrayed in their postings. Find out if your current institution has a license with the *Chronicle of Higher Education, Inside Higher Education*, or other such media sources and get into the habit of scanning them frequently. This will give insight into institutions that are thriving and on the upswing, and those in rebuilding periods. Ask scientists you meet what is it like at their institutions. When attending conferences or traveling to a new place, make an effort visit local institutions.

A mentioned in the previous section, some institutions have programs to prepare future faculty*.* These programs offer opportunities to visit the school for a mock interview and to partake in professional development. Some programs are open only to current graduate students and postdocs at the institution, but others welcome applications from outsiders. Participation in a program will provide opportunities to meet with faculty in a department or discipline of interest, view laboratories and research facilities, and explore faculty development programs. They also provide insight into the type of, and commitment to faculty professional development activities of the host institution. These programs are, in some cases, soft recruitment strategies by the host institution, whether publicly advertised as such or not.

Many institutions have revised their faculty search process to make it more equitable and accessible to PEERs (People excluded due to ethnicity or race) from the faculty job application process. However, one step in most search processes is that search committee members alert their networks, including former advisors and trainees, of the hiring opportunity. Given the historical under-representation of PEERs in advanced training and faculty positions in the U.S., recruitment through this method is likely to attract mostly non-PEER applicants. Therefore, women and PEERs should leverage their own networks to find faculty employment opportunities. Stay in touch with former advisors and mentors, and make sure they know that you are in the job market. Many scientific societies post opportunities on their websites, which often include resources specifically targeting women and PEERs. Networking is key to finding opportunities, which you might miss in a small advertisement in a journal that you might not be in the habit of reading thoroughly.

Another group that needs to take additional planning measures is those applicants who are not U.S. citizens. Most faculty openings are equally accessible to citizens and green card holders; non-citizens without a green card must consider whether the institution is willing to sponsor new faculty for visas, and for potential green card or citizenship applications. As discussed below, grant funding is critical to success and promotion of university faculty, and most federal grants require that the Principal Investigators be citizens or green card holders. For this reason, non-citizens have reduced opportunities, unless the employer is willing to sponsor the green card or application process, or the applicant has another process in place to bring about this status change.

#### Building your narrative

Every career path is different and long gone are the days when a circuitous path was viewed skeptically by search committees. Your career trajectory likely has been influenced by factors well outside your control but that you have nevertheless corralled to your advantage and success - the fact you are reading this is evidence you have navigated them successfully! How your unique path is preparing you for a faculty position at a RII is an important part of your narrative. Provide examples that demonstrate your resilience, creativity and ingenuity and other personal attributes that bode well for an academic career. Personal qualities are not easy to discern from a *cv* and you may rightly wonder if the purpose of the reference letters is precisely to “humanize” the *cv*. Take charge by creating your own narrative and sharing it with the individuals who have agreed to serve as your references. Having this information at hand would help your reviewers, if they so choose to, emphasize the elements that you feel are important in communicating the strong personal attributes you bring to the position.

Faculty search committees tend to over-emphasize quantitative metrics such as journal impact factor and engage in other practices that have been shown to be biased against individuals whose education and career trajectory has not benefitted from being in a well-funded environment such as is typical of many R1 institutions [26, 27]. In an effort to eradicate this unconscious bias search committee practices are being revised [28]. These include the adoption of a common evaluation rubric that emphasizes a criterion-based approach that supports equitable evaluation of candidates. The various components of the application dossier – cover letter, statement of teaching philosophy, and statement of commitment to diversity – provide the opportunity to personify your *cv*, and showcasing your successes in their proper context.

#### Diving in

There is no magic number for how many faculty positions one should apply for. Applying to too many can leave you exhausted and frustrated, especially if “No” letters – or, even worse, no response whatsoever to your application – begin piling early on. Applying to too few may deprive you of opportunities of weighting your options once the invitations begin to come in. Following the steps outlined in the previous sections is one way in which you can be strategic and selective. Focus on positions that you are truly passionate about, and let your passion be reflected in your application. Make allowances for a few “low-stakes” positions, maybe not your clear number 1 choices should it come decision time, but ones for which you feel your qualifications clearly exceed the expectations and there is a reasonable chance you are invited for interview. Going through the interview experience is valuable and will help you better prepare for the high stakes choices on your list. Consider that institutions take different approaches to the interview process so beware of becoming over-confident after one “successful” interview as the process may be quite different. Go to each interview with an open mind. We have seen a successful “low stakes” interview lead to a viable offer and a successful career. Don’t decide in advance which target institutions are your ideal; during the application and interview process, your opinions may change.

One common, and completely avoidable mistake made by many job prospects is to generate a single set of application material (cover letter, statement of research interest, etc) for all job applications and only change the recipient’s name. It’s inevitable, and glaringly obvious to the experienced eyes of search committee members, that such an approach leads to the presentation of material that is only remotely relevant, or not at all, to the position being applied for. For example, stating in the cover letter “I have always wanted to live in a major city” will not impress the RII in a rural setting. Neither will a cover letter that only describes your research interests impress a department where teaching is an expectation.

Each application should be personal and specific to the position described in the advertisement. Ideally, you create a boiler plate for each of the application documents describing your inalienable interests and qualities in a context sufficiently flexible that allows personalization. The responsibility of the search committee is not necessarily to select the candidate with astronomical credentials and accomplishments, but rather to select a candidate that, in their judgement, has the best chance to contribute to the program and university’s goals while developing a successful and satisfying career, and whom they see as a potential future colleague.

Develop a thoughtful and methodical approach to the application process. Start by reading the position advertisement carefully and asking a close confidant to read it as well. Is their understanding of the requirements and expectations the same as yours? Do they see you thriving in the position as well as you do? Next, study the institution, the department and its members. Can you discern patterns – e.g., years to tenure, fate of graduate program alumni, exemplary and steady leadership - that provide a picture of what is it like to be a faculty member in that department or program? As you begin the application process, create a checklist that includes all the elements of the application and key questions that you will need to address. Throughout the application process, work closely with your research advisor, peer and other mentors, and recommenders. They can offer valued critique of your application material as well as of your incoming job seminar, chalk talk or lecture. Early on, determine who is able and willing to serve in this capacity.

Common elements of a job application include the following:

#### (i) Cover letter

The cover letter should be brief and serve as a preview of the detailed information presented in the main components of your application, described below. Describe your interest and qualifications for the position and, more broadly, what attracts you about the institution and your enthusiasm for being a member of their community. Briefly address the institution’s mission, vision, strategic goals and values and how they influenced your decision to apply. Self-serving statements, such as a desire to be closer to your hometown, are not always viewed favorably and should be avoided.

#### (ii) Research statement

This statement should include your past accomplishments (briefly) and your future plans. Clearly indicate the major focus of your proposed research program, including significance of the work, specific aims and hypotheses, and potential funding sources. While this is not the time to negotiate your start-up package, do mention indispensable things you will need to succeed such as specific equipment items and collaborators. As you craft the statement, consider the interests and expertise of your potential future colleagues, who should be encouraged to see you as a collaborator. Especially if staying in a field close to your current research, make clear your plans to be independent, and not just a “postdoc at a distance” from your present lab.

#### (iii) Teaching statement

This statement should be short and concise; one to two pages unless otherwise instructed. Include teaching experience and training, building from and not simply repeating what is presented in your *cv*. Show that you have thought about how to teach, and convey your teaching philosophy and enthusiasm, commitment to student success and academic excellence, and willingness to learn. The latter is especially important if the advertisement calls for teaching a particular subject matter or in a specific modality you do not have prior experience in. Describing how you will address this potential shortcoming will avoid speculation among search committee members as to why you neglected to address a specific component of the position description. Another strategy for strengthening your teaching statement is to look at the current department course offerings; if you can develop a new course in your area of expertise that is not currently available, and you can describe how it would strengthen the curriculum, that is a plus.

#### (iv) Diversity and inclusion statement

As for the teaching statement, this statement should include a brief description of your past efforts and future activities in which you anticipate being involved. Indicate your knowledge of, enthusiasm and commitment to diversity and inclusion but do not propose to single-handedly fix all the problems or overstate your feats. Examine the institutional efforts and priorities in this critical area; be genuine in describing how you see yourself advancing the values and goals of the institution you are applying to. Do not let your race or ethnicity be the singular factor by which your candidacy will be evaluated.

#### Preparing for the interview process

Several months can go by before you are contacted for an interview. In many respects, the interview process is the most critical period of the job search and involves at least two or three stages, both virtual and in-person, over a period of months.

#### (i) Screening interview

An invitation for a screening interview indicates you are now in the “long short list”, roughly the 10–20% of candidates at the top of the applicant pool. While the odds of landing the coveted position has improved significantly, this is not the time to let the guard down. Screening interviews are almost always virtual, generally brief (approximately 30 min), include only the search committee members, and are well scripted. Committee members will take turns asking the same defined set of questions they will ask all candidates. Carefully read the invitation and prepare to answer the obvious questions, such as why the position is attractive to you. You must be able to describe your current research, future research plans (including demonstrating your independence and plans to obtain research funding), describe your experience and qualifications for future teaching, and diversity/inclusion promotion. *The opportunity to sell your candidacy must be done within the context of their questions*. Once the committee members ask their scripted questions, they will allow time for you to ask questions. While you should have a few questions ready that indicate your familiarity and interest in the position (e.g., availability of shared resources, interest in meeting specific faculty on a possible future visit), time may only allow for one or two question. Thus, it is important you not only identify the most critical question you need answered, but that you also avoid long and rambling answers to their questions as this will only take away time from your Q&A period. Follow-up questions to their questions is a good way to demonstrate your interest in the position and to begin building connectedness with the committee members. Questions about start-up, laboratory space, salary, and quality of life in the locale are not appropriate at this stage.

#### (ii) Finalist (formal) interview

Historically the formal interview has been on-campus, but at the time of this writing many institutions are conducting strictly virtual interviews. Get the logistics in advance and know what you will need prepared (and how long each should be): formal seminar, chalk talk, lecture presentation, and the people you will meet. Make a list of what you need to see, e.g., lab space, shared/core facilities, and additional people to talk to. This should include junior faculty, whom you should ask about their starting experiences, and graduate students, who generally speak with unvarnished and unapologetic candor. You will play the role of interviewee and interviewer – you should ask as many questions of them as they will ask of you. Admittedly, the in-person interview can be an exhausting experience that often involves one to 3 days of back-to-back meetings and talks with you as the main attraction – a marathon version of speed dating. You must be prepared for this test of your stamina and maintain an even-keel level of enthusiasm and interest in the work and opinions of everyone you meet. This task will be less onerous for the well-prepared: those that have gathered information, such as research and teaching interests, years and position at the institution, for everyone listed in the interview agenda. Be prepared to be in command of the conversations, this will not only mitigate anticipatory anxiety but also contribute to a most productive experience. Remember, everyone who meets you is an interviewer, so engage positively with students, staff, and local drivers (who may be students or junior faculty).

In addition to learning about the people, their work and experience at the institution, the facilities and the program, you should walk away from the interview with a general understanding of the policies, processes and procedures that will govern the conditions of your appointment. Ask for documentation of promotion and annual evaluation guidelines, department strategic plan, or academic and research goals. Unless someone volunteers to provide the information (or, it is not easily available through a web search), you should avoid requesting specific information about salary, benefits, and vacation time. It is also not the time to inquire about a position for your partner or spouse. Lastly, once you have departed, demonstrate your appreciation and continued interest by sending a brief thank you note (email is fine!) to everyone you met. You will make an indelible mark in the search committee members and department chair if you share your interview impressions – how you now see yourself contributing to the department’s research and teaching mission. Regarding the latter, you can mention specific assignments in which you would be interested, especially if they complement and expand their current course offerings.

#### (iii) Offer and site visit

Once you are offered a position, you may request, or be offered, a second visit (or first, if the finalist interview was conducted virtually) to further explore your options, scrutinize facilities, examine and plan your lab and office space, and check out the campus and vicinity, among others. Though you may have been made a verbal offer and the visit is informal, this is once again not the time to let the guard down. Treat the visit as if the prospect is still in the balance. By now you may have shared a start-up list with the department chair or direct supervisor; it is imperative that you satisfy your major equipment needs by the end of the visit. By now you should have also started negotiations regarding salary and, if applicable, a position for your partner or spouse.

Find out what recent new hires have received in salary and start-up while keeping in mind that their research and equipment needs may vary vastly from yours. You don’t have to get the same package as the last hire, but don’t go into a new job without access to the essential equipment you’ll need to succeed. Salary is influenced by several factors, including type of institution (e.g., private versus public) and the local cost of living. You’ll need more in New York or San Francisco than in a small town in the Mid-West. It is helpful to know the range of and mean salaries for your discipline in the type of institution and region and this information is available through several sources [29, 30]. If your offer is at a public institution, individual salary information is readily available. Finally, ask what assistance the employer can provide in identifying housing, school, house of worship, employment for your spouse or partner if outside academia, etc.

Start-up packages at an RII can easily exceed several hundred thousand and well into the $1–3 million range. This seems very generous, but may have to cover renovations, core facility use charges, staff salaries and student stipends, your summer salary (for 9 month appointments) and the purchase and maintenance of equipment, in addition to the cost of reagents and expendable supplies. As you discuss start up packages, make sure you know what will have to be included as part of the package, any restrictions or conditions that may apply, and how long you will have to expend the funds. A three-year period is typical.

There are many other elements to the site visit and offer negotiation. For additional guidance, we refer you to the article by Goldstein and Avasthi in this volume [31].

#### Navigating the transition

The period from accepting the offer to starting your new faculty position can extend for several months. Some schools may follow a somewhat scripted onboarding plan that facilitates this transition. This plan may include multiple individuals with assigned tasks – from the department administrator who sets up the start-up budget and other employment forms, the department chair who fosters development of personal and professional relationships with members of the department, university, and community, to the associate dean or vice provost who oversees the professional development framework. More often, you will need to anticipate a strategy and work with your contacts at the new institution as well as with your professional network to effectuate an as seamless as possible transition. An early start to the process of setting up your lab and becoming familiar with the personnel and resources at your institution can go a long way towards ensuring a productive and rewarding career. The following, while not intended to represent a comprehensive list, are particularly important:

#### (i) Setting up the lab

If your research space will require renovations, work with your university contact to begin them as soon as possible. Depending on the extent and type of renovations there may be multiple campus units and individuals involved (e.g., architectural design, physical plant, risk management) in addition to outside contractors in the case of specialized equipment or needs. Ask your chair or department administrator to identify a point person for the project and communicate with them as often as needed, at least once a week. You will need engineering controls and certification for the space as well as for your personnel if your work involves biohazards and chemical hazards. You should ask to be provided with the guidelines and regulations pertaining to your type of research.

Your chair and department administrator, as well as your new colleagues, can provide essential advice on securing laboratory equipment and supplies. Many universities require at least two quotes from different suppliers for the purchase of equipment above a certain price range. Ask for information about the purchasing method at the institution as well as vendors with whom they may have contracts. If your lab space is undergoing renovation or otherwise inaccessible, work with the department administrator to identify a safe space to store the equipment and supplies, and ask to be notified every time a shipment arrives. The article by Goldstein and Avasthi offers additional advice on purchasing and excellent advice on recruiting and onboarding lab personnel [31].

#### (ii) *Workload and professional development*

As discussed earlier, your responsibilities as a faculty member will fall mostly into three categories – research, teaching and advising, and service. The percentage of the workload effort that is assigned to each of these categories varies from institution to institution, and from year to year, and there is often room for negotiation. At most RIIs, it is not uncommon for teaching and service to be light the first year but expect these efforts to increase. Your focus will be on getting the lab off the ground and securing funding. If there will be a long lead-time until your new lab and equipment are ready, you might ask that this light load be delayed until your second year. Develop a plan that allocates time proportionally to your workload percentages, e.g., if service accounts for 5% of your workload plan to dedicate approximately 2 h per week. In addition, find out if your institution offers some version of a junior leave – a semester with reduced or no teaching prior to the tenure review to provide focused time for research, publications or grant proposal submission. Having this information early on will better serve you in laying out a multi-year strategy.

## Conclusion

The transition to a faculty position is an ideal time to refresh your mentoring network and cultivate sponsors and collaborators. Announcing that you have secured a faculty position is certainly newsworthy and is a vehicle for creating new contacts on social networks. Ask the department chair and new colleagues at your new institution to introduce you to the contact person in key offices such as sponsored programs and faculty professional development units. Most likely, you will meet many of these individuals and the services they provide at a new faculty orientation at the beginning of your employment. Avail yourself of resources that foster your professional and personal growth and work with your designated mentor (or, ideally, a personal board of advisors [32]) in planning your immediate and long-term goals.

New faculty who are PEERs or women, may find themselves being “solo” or one of the few faculty in the department from their demographic. This may tempt the chair to over-burden such faculty with service on multiple committees addressing diversity or inclusion, or to appoint them to many committees to “diversify” those groups. While service is required, as a junior faculty you will need time to write grants, prepare for teaching, and do research. If you are considering a department where you will be the first from your group, make sure that the chair understands that you will need protection from being assigned too much committee work and that your mentoring needs will differ from those of non-PEER and male colleagues.

A final bit of advice: Don’t get discouraged by rejection letters or lack of response. These do not necessarily indicate any lack of merit. Each department is likely hiring only one new faculty at any time, and individual perceptions of how each candidate fits into the specific niche to be filled generally outweigh any consideration of absolute merit! Keep going until you find the position for which you are right, and which is right for you.

## Data Availability

Not a data-based article.

## Abbreviations

ASCB: American Society for Cell Biology
*CV*: Curriculum vitae
PEER: People excluded due to ethnicity or race
PUI: Primarily undergraduate institution
Q&A: Question and answer
RII: Research intensive institution
STEM: Science, technology, engineering and mathematics
URM: Underrepresented minority
US: United States of America
